# Chronic Fatigue, Physical Impairments and Quality of Life in Women with Endometriosis: A Case-Control Study

**DOI:** 10.3390/ijerph17103610

**Published:** 2020-05-21

**Authors:** Francisco Álvarez-Salvago, Ana Lara-Ramos, Irene Cantarero-Villanueva, Maryna Mazheika, Antonio Mundo-López, Noelia Galiano-Castillo, Carolina Fernández-Lao, Manuel Arroyo-Morales, Olga Ocón-Hernández, Francisco Artacho-Cordón

**Affiliations:** 1 “Cuídate” Support Unit for Oncology Patients (UAPO), Sport and Health University Research Institute (iMUDS), University of Granada, E-18016 Granada, Spain; salvagofran@gmail.com (F.A.-S.); irenecantarero@ugr.es (I.C.-V.); noeliagaliano@ugr.es (N.G.-C.); carolinafl@ugr.es (C.F.-L.); marroyo@ugr.es (M.A.-M.); 2 Department of Physiotherapy, University of Granada, E-18016 Granada, Spain; 3 Gynaecology and Obstetrics Unit, “Virgen de las Nieves” University Hospital, E-18014 Granada, Spain; ana_lara_ramos@hotmail.com; 4 Biohealth Research Institute in Granada (ibs.GRANADA), E-18016 Granada, Spain; ooconh@ugr.es; 5 Department of Obstetrics and Gynaecology, University of Granada, E-18016 Granada, Spain; marinamazheika@ugr.es; 6 Centro de Psicología Clínica Alarcón (CPCA), E-18012 Granada, Spain; antonmundol@hotmail.com; 7 Gynaecology and Obstetrics Unit, “San Cecilio” University Hospital, E-18016 Granada, Spain; 8 Department of Radiology and Physical Medicine, University of Granada, E-18016 Granada, Spain; 9 CIBER of Epidemiology and Public Health (CIBERESP), 28015 Madrid, Spain

**Keywords:** chronic fatigue, endometriosis, health-related fitness, health-related quality of life, sleep quality

## Abstract

Aim: To explore endometriosis-related fatigue (ERF), health-related fitness, sleep quality, and health-related quality of life (HRQoL) in women with endometriosis in comparison with matched controls. Methods: Twenty-five affected women and twenty-five age and height-matched women without endometriosis were included. ERF was assessed through the Piper Fatigue Scale; health-related fitness was assessed through the Schöber, flamingo, and 6-min walking tests and dynamometry; and body composition was assessed through impedanciometry. Self-perceived physical fitness, sleep quality, and HRQoL were assessed through the International Fitness Scale, the Pittsburgh Sleep Quality Index, and the 12-item Short Form Health Survey, respectively. Results: Affected women exhibited higher levels of ERF than controls, increased fat mass, and physical deconditioning (reduced back strength, lumbar flexibility, body balance, and functional capacity, *p*-values < 0.050). Moreover, cases also had poorer perceived physical fitness, sleep quality, and HRQoL (*p*-value < 0.050). Finally, we observed deteriorated health-related fitness, sleep quality, and HRQoL in those women with endometriosis with higher levels of ERF. Conclusions: This study constitutes the first evidence that women with endometriosis describe a generalized physical deconditioning, even more pronounced in affected women with higher levels of ERF. Further studies assessing the efficacy of rehabilitation interventions to face these physical impairments in women with endometriosis are warranted.

## 1. Introduction

Endometriosis is a common gynecological disorder affecting women of reproductive age, which is characterized by the ectopic proliferation of endometrial-like tissue outside the uterine cavity [1], with both genetic and environmental factors associated with its development [2]. Despite the absence of national networks of endometriosis registries and the occurrence of asymptomatic cases of endometriosis, it is estimated that 2–15% of women in childbearing age are affected worldwide, rising up to 30% among infertile women and up to 45% among women with chronic pelvic pain [3]. Besides the absence of accurate noninvasive diagnostic tools, current surgical and medical treatments for endometriosis (e.g., surgical ablation of some endometriotic lesions or oral contraceptives and analgesics [1]) are focused on the amelioration of main symptoms and complications but not on achieving the complete disappearance of the disease. Even so, current treatments usually fail to manage endometriosis symptoms [4]. The absence of definitive curative treatments leads to considering this disease as a chronic and progressive condition [5], which has been described to be a risk factor for miscarriage [6], infertility [1], and mid/long-term gynecological cancer [7]. Moreover, it has been reported the debilitating condition of endometriosis [8,9]. Apart from the well-acknowledged chronic pelvic pain experienced by almost all affected women, which is usually enhanced during the menstruation period or during the accomplishment of some activities of daily living, such as defecation, urination, or sexual intercourse [10], there is a lack of studies that specifically assess the physical performance of these women with endometriosis. Until now, only a few studies have pointed out that these women also experience chronic fatigue [11,12,13] that significantly causes distress in these women [14]. In fact, it has been estimated that this endometriosis-related fatigue (ERF) might be frequent in more than a half of the affected women [11]. Moreover, they also found that ERF was associated with insomnia, depression, pain, and occupational stress [11].

However, there is a lack of information regarding other aspects of the physical performance of women with endometriosis. Until now, some studies have reported physical deconditioning in different subgroups of patients with chronic diseases such as cancer survivors [15], those with fibromyalgia [16], or those with low back pain [17]. Thus, we hypothesized that women with endometriosis would show reduced physical performance, which seems to be a relevant factor on daily living physical demands and quality of life. For that, the aim of the present study was to explore ERF, health-related fitness, sleep quality, and health-related quality of life (HRQoL) in women with endometriosis compared with matched controls.

## 2. Material and Methods

### 2.1. Subjects

Between January 2018 and January 2019, 25 women diagnosed with endometriosis were recruited from the Gynecology and Obstetrics units of both “San Cecilio” University Hospital and “Virgen de las Nieves” University Hospital, Granada, Spain. To be eligible for this observational case-control study, women had to be between 25 and 50 years, to have a clinical diagnosis of endometriosis, to suffer from endometriosis-related symptoms, and to have passed a period equal to or greater than three months since the last surgery endometriosis (ENDO) group. Participants were excluded if they had a diagnosis of any concomitant debilitating musculoskeletal or autoimmune disease and any medical condition or other reasons that did not allow participants to read or perform the assessment.

Volunteers who responded to a university announcement were selected as age and height-matched controls. Given the plausibility of non-symptomatic endometriosis cases, controls additionally underwent a gynecological examination and a transvaginal ultrasonography by a trained gynecologist to ensure that controls were asymptomatic and without ultrasound-visible endometrial lesions. Furthermore, control group participants were excluded from this study if they presented history of endometriosis-related symptoms or infertility, presented severe previous comorbidities, as well as had undergone surgery in the last three months.

Epidat 3.4 software (Xunta de Galicia, Santiago de Compostela A Coruña, Spain) was used to estimate the required sample size. It was calculated to detect a minimal difference of three points in the fatigue total score. In the absence of previous studies assessing fatigue in endometriosis women with a validated scale when this study was conceived, this difference was selected based on a previous study in breast cancer survivors, another chronic female condition with high levels of chronic fatigue and physical impairments that considered this three-point difference a mild level of fatigue [18]. Hence, with an α-level of 0.05, a desired power of 90%, and an estimated standard deviation of 3.0 points, we needed a total of 22 participants for each group. Considering a 10% dropout rate, we finally included a total of 25 participants for each group.

Participants with endometriosis and matched healthy controls signed informed consent forms prior to being enrolled in the study, which followed the Helsinki Declaration for biomedical research and was approved by the Ethical Committee on Biomedical Research of Granada (CEIm) (0792-N-18).

### 2.2. Assessment

Participants were assessed for one hour approximately by a trained physiotherapist, and in order to minimize the influence of physiological discomfort related to menstrual cycle on the study results, all the evaluations took place between the 2nd and the 10th day after menstruation in women who were not using hormonal contraceptives, although no differences in pressure pain thresholds have been previously reported [19].

#### 2.2.1. Endometriosis-Related Fatigue (ERF)

The level of fatigue was determined with the Spanish version of the Piper Fatigue Scale (PFS) [20]. The PFS contains 22 items for which the scores range from 0 to 10 and includes four dimensions of subjective fatigue: behavioral/severity, affective meaning, sensory, and cognitive/mood. The total fatigue score is calculated with higher scores indicating higher levels of fatigue. The PFS, with high reliability (Cronbach’s α > 0.86) [20,21], has been widely used in a variety of chronic female diseases such as breast cancer and gynecological disorders [22,23] as well as other musculoskeletal conditions [24].

#### 2.2.2. Health-Related Fitness Outcomes

A back dynamometer (TKK 5002 Back-A, Takey, Tokyo, Japan) with a precision of 1 kg was used for the measurement of isometric back strength, which has revealed acceptable to good reliability (intraclass correlation coefficient (ICC) ranged between 0.81 and 0.85)) [25]. For that, women in a standing position, fully extended knees, and a lumbar flexion of 30° were asked to perform an extension of the trunk. The average value of three trials (1-min intertrial delay) was used for the analyses.

A digital dynamometer (TKK 5101 Grip-D, Takey, Tokyo, Japan) with a precision of 0.1 kg was used to assess the upper body muscular strength in women in a bipedal position and the arm in complete extension. To determine the optimal grip span according to hand size, a validated algorithm was used [26]. The average value of three attempts (1-min intertrial delay) from each hand was used for analyses. This test has shown to be valid and reliable [27].

Lumbar spine flexibility was evaluated with the Original Schöber test [28]. Participants had to be in a bipedal position, and marks were made on the lumbosacral junction and 10 cm above the first mark. Participants were asked to bend forward as far as possible, keeping their legs straight. The new distance between the two marks was recorded as maximum flexion. Higher distances during flexion represent better flexibility. This test has previously shown to be reliable (ICCs 0.85–0.96) [29].

Body balance was determined using the Flamingo Test. The participants stood on a beam with their shoes removed. While balancing on the preferred leg, the free leg was flexed and the knee and the foot of this held leg were close to the buttocks. The number of trials needed to complete 30 s of the static position was recorded, and the chronometer was stopped if the participants did not comply with the protocol conditions. Lower flamingo balance scores indicate a better whole-body balance. The average of both legs was used in the analysis. This test has shown to be valid and reliable with an ICC of 0.71 [30].

Functional capacity was evaluated using the 6-min walk test, which determines the maximum distance (in meters) that women are able to walk along a 30-m linear circuit for 6 min [31]. This test showed adequate reliability (ICC = 0.74) [32].

#### 2.2.3. Self-Reported Physical Fitness, Sleep Quality, and Quality of Life

The physical fitness was assessed through the Spanish version of the International Fitness Scale (IFIS) [33], a simple and short self-administered scale consisting on a 5-point Likert scale with 5 responses (“very poor”, “poor”, “average”, “good”, and “very good”), with higher scores representing better perceived physical fitness. This scale addresses patients perceived overall fitness, cardiorespiratory fitness, muscular fitness, speed-agility, and flexibility. IFIS has shown good reliability (Cronbach’s alpha > 0.80) [34].

The sleep quality was evaluated with the Spanish version of the Pittsburgh Sleep Quality Index (PSQI) [35]. It is a 19-item, validated, self-report scale that measures quality and patterns of sleep. Scores range from 0 to 21, with higher scores indicating poorer sleep quality. The PSQI has shown good reliability (Cronbach’s alpha 0.87) [36].

The Spanish version of the SF-12 was used to evaluate the HRQoL, which is a shortened form of the SF-36 questionnaire [37]. It consists of twelve questions that measure physical and mental health. Each of the components are scored on a scale from 0 to 100, with higher scores indicating better health-related quality of life. The SF-12 has shown high reliability with an ICC ranged between 0.64 and 0.73 [38].

#### 2.2.4. Body Composition

Regarding body composition, height was measured with an inelastic tape, while weight, body mass index (BMI), skeletal muscle mass, and percentage of body fat were obtained with a bioelectrical impedance analysis (InBody 720, Biospace, Seoul, South Korea), which has demonstrated good reliability with an ICC > 0.98 [39].

### 2.3. Data Analysis

We used the Statistical Package for the Social Sciences (IBM SPSS Statistic for Windows, Armonk, NY, USA version 23.0) with a 5% level of significance. Given the limited sample size (*N* ≤ 50) and the non-normal distribution of variables (tested with the Kolmogorov–Smirnov test), the Mann–Whitney U and Chi-square tests were used to examine differences between groups for continuous and categorical variables, respectively.

The main analysis was tested using the Mann–Whitney U test. The groups served as an independent variable (ENDO group or control group), and all ERF, health-related fitness (strength, flexibility, body balance, functional capacity, and body composition), self-reported physical fitness, sleep quality, and quality of life were used as dependent variables. The contribution of fatigue to health-related fitness, self-reported physical fitness, sleep quality, and HRQoL was assessed with the Jonckheere–Terpstra trend test after stratification of the ENDO group according to the median PFS score. No imputation techniques were necessary given the absence of missing values.

## 3. Results

Characteristics of the groups were summarized (Table 1)**.** No differences were observed in the sociodemographic characteristics of the 50 participants (including age, educational level, or cohabitation) with the exception of employment status, with higher rate of unemployment in the ENDO group (*p*-value = 0.004). Concerning body composition, women in the ENDO group showed an increased percentage of body fat mass with respect to the control group (35.7% ± 8.9% vs. 28.2% ± 7.2%, *p*-value 0.007). Similarly, the ENDO group showed increased BMI (26.4 ± 6.1 vs. 23.0 ± 2.6 kg/m^2^) and weight (70.8 ± 15.9 vs. 62.4 ± 9.0 kg), although these differences did not reach the statistical significance (*p*-values 0.054 and 0.075, respectively). Regarding pelvic pain intensity during the examination, all controls reported absence of or mild pelvic pain, while 14 (56.0%) out of 25 women with endometriosis reported mild pelvic pain and 11 (44.0%) affected women reported moderate/severe pelvic pain (data not shown in tables).

### 3.1. Chronic Fatigue

**Figure 1:**
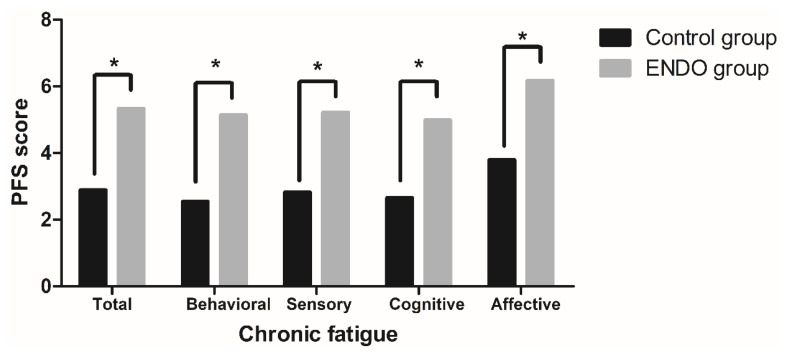
Levels of endometriosis-related fatigue (ERF) in women with (ENDO group) and without endometriosis (control group). PFS: Piper Fatigue Scale. *p*-values were calculated through the Mann–Whitney U. *p - value < 0.050.

Levels of ERF are summarized in Figure 1. The Mann–Whitney U test results revealed that total PFS score as well as the scoring of all of PFS dimensions were significantly higher in endometriosis women than in controls (*p*-values < 0.001), with a median total fatigue score of 5.3 ± 2.3 in the ENDO group and 2.9 ± 2.0 in the control group. Additionally, in order to exclude the potential effect of BMI levels on the differences found, we have conducted subanalyses comparing (i) control women (20 (80.0%)) and women from the ENDO group (10 (40.0%)) in the normal range of BMI (normal weight, BMI < 25.0 kg/m^2^) and (ii) women with normal weight (10 (40.0%)) vs. women with overweight/obesity (15 (60.0%)) from the ENDO group. As shown in Table 1, levels of fatigue were significantly higher in women with endometriosis than in controls when only women with normal weight were considered (5.16 ± 2.01 vs. 2.92 ± 2.05, *p*-value 0.003), while levels of fatigue were not significantly different between women with overweight/obesity and normal weight within the ENDO group (4.80 ± 2.29 vs. 5.16 ± 2.01, *p*-value 0.598). For further analyses assessing the influence of fatigue in health-related fitness and patient-reported outcomes, ENDO group participants were categorized according to the PFS median value (5.5): low-fatigued (≤5.5, *n* = 13) and fatigued (>5.5, *n* = 12).

### 3.2. Health-Related Fitness

Results for the health-related fitness measurements are depicted in Table 2. The Mann–Whitney U test results revealed significant differences between groups in the isometric back strength but not in the strength of the upper body. Hence, back strength values obtained in the ENDO group were lower than in the control group (52.2 ± 14.7 vs. 63.2 ± 11.4 kg, *p*-value 0.006). Regarding flexibility, the ENDO group had lower lumbar flexibility than controls (14.1 ± 1.1 vs. 15.0 ± 1.1 cm: *p*-value 0.011). Finally, significant differences between groups were observed for body balance and functional capacity. In this sense, endometriosis women showed worse body balance, i.e., higher score in the flamingo test (0.2 ± 0.3 vs. 0.0 ± 0.1; *p*-value 0.021), and shorter walked distance (561.2 ± 57.9 vs. 651.2 ± 54.3 m, *p*-value < 0.001) compared with the control group.

In the subanalyses conducted according to BMI, significant differences were found in lumbar spine flexibility, body balance, and functional capacity between controls with normal weight and women with endometriosis with normal weight, with poorer scores in the latter group (*p*-values < 0.050). The reduced back strength found in women with endometriosis with normal weight compared to controls was close to the statistical significance (*p*-value 0.054). Additionally, comparisons between the healthy group and both the low-fatigued (≤5.5) and the fatigued (>5.5) ENDO subgroups showed that isometric back strength was significantly lower in endometriosis women with high fatigue (51.0 ± 17.4 kg) than in low-fatigued endometriosis women (53.3 ± 12.2 kg) and the healthy group (63.2 ± 11.4 kg) (*p*-trend 0.022) (Supplementary Table S1). Similarly, lumbar flexibility was sequentially reduced in low-fatigued (14.3 ± 0.9 cm) and high-fatigued patients (14.0 ± 1.2 cm) in comparison to controls (15.0 ± 1.1 cm) (*p*-trend 0.030). Functional capacity was also serially decreased in low-fatigued (571.9 ± 65.5 m) and high-fatigued endometriosis patients (549.8 ± 48.5 m) in comparison with the control group (651.0 ± 54.3 m) (*p*-trend < 0.001).

### 3.3. Self-Reported Physical Fitness, Sleep Quality, and Quality of Life

Results from the self-reported physical fitness, sleep quality, and HRQoL are summarized in Table 3. The Mann–Whitney U test did find significant differences between groups in the level of perceived overall physical fitness, cardiorespiratory fitness, muscular fitness, and speed-agility, with better scores in the control group (*p*-values < 0.05). Similarly, significant differences between groups in sleep quality were found. In this sense, women in the ENDO group exhibited a higher total score (*p*-value = 0.017), indicating poorer sleep quality than those in the control group. Finally, the Mann–Whitney U test results revealed that the HRQoL was significantly different between groups. In this respect, the ENDO group exhibited lower scores in the physical health domain, indicating a worse HRQoL (*p*-value < 0.001) in comparison with the control group. Notwithstanding, no significant differences between groups in the mental health domain were found.

Analyses stratified by BMI category revealed significant differences between groups. Lower scores were obtained in sleep quality and in the SF-12 physical health domain in women with normal weight from the ENDO group than in controls with normal weight, while no differences were observed between women with normal weight and overweight/obesity within the ENDO group (Table 1). Finally, poorer perceived overall physical fitness, cardiorespiratory, and speed-agility as well as sleep quality were found in low-fatigued (≤5.5) and fatigued (>5.5) women from the ENDO group and controls. Moreover, those fatigued women from the ENDO group showed lower scores than those women from the low-fatigued ENDO subgroup and the healthy group in HRQoL (physical domain) (Supplementary Table S2).

## 4. Discussion

To the best of our knowledge, this study is among the very first to offer a complete characterization of the health-related fitness in women with endometriosis. The main finding of this study is a significant physical deconditioning in affected women compared with the control group. Hence, our findings indicate that women with endometriosis had lower back strength, lumbar flexibility, body balance and functional capacity. Moreover, affected women perceived lower physical fitness and poorer sleep quality and HRQoL. Finally, we found that women with endometriosis experienced ERF that negatively influenced all the health-related fitness, sleep quality, and HRQoL outcomes.

We have found higher levels of ERF reported by women in the ENDO group with respect to women from the control group. Our results are in line with those reported previously [11] that found that 77.8% of affected women experience occasional/frequent fatigue, although they did not assess ERF with a validated ordinal scale. Similarly, Surrey, et al. [40] reported that the percentage of women with some degree of fatigue was greater than 60%. In our study, we found that fatigue level was significantly higher in women with endometriosis, with moderate and severe ERF present in 13 (52.0%) and 7 (28.0%) of the affected women, respectively. In this regard, although some previous publications have shown that both surgical and medical treatments might help to reduce ERF levels in some patients [40,41], we have observed that moderate/severe ERF is present in the majority of women with endometriosis that have confirmed full adherence to medical treatment and in those that have undergone previous surgical interventions.

Muscle strength is crucially related to the easiness to face daily living physical demands. Low back muscles are involved in loads imposed on the lumbar spine during activities of daily living related to manual handling of materials during all household, worktime, and leisure time [42]. In this regard, we have found that strength of low back muscles in women with endometriosis was 17.4% lower than in women from the control group. Although no previous studies have reported this difference in muscle strength in women with endometriosis, it could be explained, at least in part, by the local pain experienced by these women in this body area, which may lead to a reduced activity in trunk muscles. In fact, our results are in line with those found in other similar chronic conditions such as ankylosing spondylitis [43] or low back pain [44]. Similarly, we have found that this part of the spine also showed reduced flexibility in the ENDO group. Despite the scarcity of previous studies assessing this outcome in women with endometriosis, it seems plausible that adhesions created by endometriotic lesions in the abdominal area may partially explain the observed hypomobility in this spinal region. Moreover, endometriosis-related pain and the thickness of lumbar fascia attached to lumbar vertebrae [45] may also contribute to restrictions in joint range of motion [46]. Additionally, women from the ENDO group showed lower postural balance, in line with findings from other studies assessing physical fitness in different patient subpopulations with chronic diseases affecting the lumbopelvic area, such as colorectal cancer survivors [15] or those with chronic low back pain [47,48], where the authors reported abnormal spinal proprioception and lower body balance. In addition to muscle impairments that can originate a poor position sense [49], sensory, biomechanical, and motor-processing strategies are required for an adequate static balance [50] and are even more complex for an efficient movement function and maintenance of balance during dynamic tasks [50]. Moreover, deficits in either static or dynamic balance are associated with an impaired ability to cope with daily living activities [51]. Finally, a poorer functional capacity was found in women from the ENDO group, with a 13.8% reduction in comparison with matched controls and a 5.5% decrease in comparison to previously estimated distances in healthy adult women [52]. Similar reductions were found in women with chronic illnesses such as fibromyalgia [53].

In addition to poorer scores in all health-related fitness components, the ENDO group also perceived lower physical fitness in addition to poorer sleep quality and HRQoL. These findings are in agreement with many studies that have previously reported worse scores in HRQoL [54,55,56,57,58] and sleep quality indices in women with endometriosis [55,59]. However, this study constitutes the first report of lower self-reported physical fitness in women with endometriosis. Despite the novelty of this finding, it is in agreement with other studies involving chronic patients such as cancer survivors [15] or fibromyalgia [60], among others.

Another interesting finding of this study is that those women in the ENDO group with higher levels of ERF (above the median) showed a significant reduction in almost all the components of the health-related fitness (including back strength, lumbar flexibility, body balance, functional capacity, and fat mass) as well as self-reported physical fitness, sleep quality, and HRQoL.

This study has some limitations that should be considered before extrapolating the findings reported. Firstly, although the cross-sectional design of this study allowed us to describe differences in the physical status of affected women in comparison with matched controls, the onset or trajectory over time of physical function of women with endometriosis remains unmeasured. Secondly, the limited number of affected women considerably reduced the statistical power of subanalyses performed across patients. However, we satisfactorily identified that ERF is a relevant aspect that may influence the physical status and the HRQoL. Moreover, other parameters not considered in this study, such as endometriosis-unrelated postsurgical adhesions in cases or controls, might be involved in the associations found. In addition, despite the confirmation of the absence of endometriosis-related symptoms and ultrasound-visible endometrial lesions by a trained gynecologist in control women before examination, we cannot fully rule out the presence of any ultrasound-invisible endometrial lesion in any control women. Finally, stage-dependent differences in the study outcomes have not been accomplished given the limited size of the sample. Therefore, these promising results warrant further studies assessing potential differences in health-related fitness according to the stage of endometriosis or the number of surgeries that patients have undergone.

This study has relevant clinical implications. Endometriosis and endometriosis-related symptoms could lead to a global deconditioning process including strength, lumbar flexibility, resistance, and balance components that may lead to significant impairments during the performance of activities of daily living, including household, work, and leisure tasks. Thus, tailored rehabilitation programs, such as therapeutic exercise interventions targeting several aspects of fitness, may help to reduce ERF and to improve quality of life in women with endometriosis.

Taken together, these findings strongly support the presence of moderate/severe ERF in most of women with endometriosis, a perceived and objective decrease of physical fitness, and a reduction in sleep quality and the HRQoL, which were even more pronounced in those affected women with higher ERF levels. Thus, further studies assessing (1) stage-dependent differences in physical status and (2) efficacy of exercise-based intervention programs are warranted.

## 5. Conclusions

This study indicates that women with endometriosis usually experience chronic fatigue and has a decrease of physical fitness. Particularly, compared to healthy controls, affected women had lower back strength and lumbar spine flexibility, as well as reduced body balance and functional capacity. Interestingly, this study reveals that this decrease of physical fitness is associated with the ERF levels exhibited by affected women. Thus, this study warrants the evaluation of exercise-based intervention programs that target these aspects related to ERF and physical fitness of women with endometriosis.

## Figures and Tables

**Table 1:** Characteristics of the study population (*N* =50).

Variables	Control Group *(N = 25)*	ENDO Group *(N = 25)*	*p-*Value
	*n* (%)	*n* (%)	
**Age** (yrs) *	34.5 ± 5.2	36.2 ± 3.7	0.150
**Schooling**			0.185
*University*	21 (84.0)	17 (68.0)	
*Up to high school*	4 (16.0)	8 (32.0)	
**Cohabitation**			0.333
*Living alone*	8 (32.0)	5 (20.0)	
*Living as a couple*	17 (68.0)	20 (80.0)	
**Employment**			**0.004**
*Working outside*	23 (92.0)	14 (56.0)	
*Not working outside*	2 (8.0)	11 (44.0)	
**Endometriosis diagnosis**			-
*Laparoscopy*	-	19 (76.0)	
*MRI*	-	6 (24.0)	
**ASRM staging**			-
*I-III*	-	7 (28.0%)	
*IV*	-	18 (72.0%)	
**Endometriotic lesion location**			-
*Deep infiltrating endometriosis*	-	7 (28.0%)	
*Ovarian/peritoneal endometriosis*	-	18 (72.0%)	
**Endometriosis surgical interventions**			-
*None*	-	6 (24.0)	
*One*	-	11 (44.0)	
*Two or more*	-	8 (32.0)	
Body composition *			
*Height (m)*	164.0 ± 7.9	163.7 ± 5.5	0.796
*Weight (kg)*	62.36 ± 8.97	70.76 ± 15.87	0.175
*BMI (kg/m^2^)*	22.98 ± 2.55	26.39 ± 6.05	0.054
*Lean mass (kg)*	24.05 ± 2.88	24.27 ± 3.39	0.920
*Fat mass (%)*	28.23 ± 7.16	35.73 ± 8.93	**0.007**

* Mean ± standard deviation; MRI: magnetic resonance imaging; BMI: body mass index. ASRM: American Society for Reproductive Medicine. Bold numbers indicate significant differences between groups.

**Table 2:** Health-related fitness in women with and without endometriosis.

Outcome	Control Group (*n* = 25)	ENDO Group (*n* = 25)	*p-*Value
**Strength**			
*Back dynamometer*	63.20 ± 11.40	52.20 ± 14.70	**0.006**
	(58.26–68.12)	(46.17–58.28)	
*Hand dynamometer, dominant side*	26.50 ± 4.00	26.10 ± 4.70	0.712
	(24.53–27.46)	(24.18–28.07)	
*Hand dynamometer, nondominant side*	25.20 ± 3.90	24.10 ± 5.00	0.295
	(23.35–26.01)	(22.04–26.18)	
**Lumbar spine flexibility**			
*Schöeber test (cm)*	15.00 ± 1.09	14.10 ± 1.06	**0.011**
	(14.48–15.46)	(13.70–14.58)	
**Body balance**			
*Flamingo test*	0.02 ± 0.10	0.18 ± 0.32	**0.021**
	(−0,02–0.07)	(0.05–0.31)	
**Functional capacity**			
*6-min walking test (m)*	651.20 ± 54.30	561.20 ± 57.90	**<0.001**
	(624.42–672.71)	(537.35–585.13)	

Values are expressed as mean ± standard deviation (95% confidence intervals for the mean). Bold numbers indicate significant differences between groups.

**Table 3:** Perceived physical fitness, sleep quality, and quality of life in women with and without endometriosis.

Outcome	Control Group (*n* = 25)	ENDO Group (*n* = 25)	*p-*Value
**Physical fitness**			
*Overall fitness*	3.60 ± 0.65	2.74 ± 0.81	**<0.001**
	(3.31–3.86)	(2.39–3.09)	
*Cardiorespiratory fitness*	3.25 ± 0.79	2.57 ± 0.90	**0.011**
	(2.91–3.59)	(2.18–2.95)	
*Muscular fitness*	3.63 ± 0.71	3.09 ± 0.85	**0.032**
	(3.32–3.93)	(2.72–3.45)	
*Speed-agility*	3.71 ± 0.75	2.96 ± 0.56	**<0.001**
	(3.39–4.03)	(2.71–3.20)	
*Flexibility*	3.38 ± 0.77	3.13 ± 1.06	0.408
	(3.05–3.70)	(2.67–3.59)	
**Sleep quality**			
*Pittsburgh Sleep Quality Index*	6.00 ± 3.19	8.32 ± 3.72	**0.017**
	(4.68–7.32)	(6.79–9.85)	
**HRQoL**			
*Physical health*	55.09 ± 5.04	37.99 ± 13.08	**<0.001**
	(53.01–57.17)	(31.68–44.29)	
*Mental health*	49.56 ± 10.54	46.36 ± 11.66	0.271
	(45.21–53.91)	(40.74–51.99)	

Values are expressed as mean ± standard deviation (95% confidence intervals for the mean). HRQoL: health-related quality of life. Bold numbers indicate significant differences between groups

## Supplementary Materials

The following are available online at https://www.mdpi.com/1660-4601/17/10/3610/s1, Table S1: Health-related fitness in healthy women and affected women with low fatigued and fatigued, Table S2: Perceived physical fitness, sleep quality and quality of life in healthy women and affected women with low fatigued and fatigued.
