# Supplementary material for: Chronic Fatigue, Physical Impairments and Quality of Life in Women with Endometriosis: A Case-Control Study
Source: Int J Environ Res Public Health. 2020 May 21;17(10):3610. doi: 10.3390/ijerph17103610 (PMC7277433; doi:10.3390/ijerph17103610)
Supplement: Supplementary file 1 [file ijerph-17-03610-s001.pdf]

Supplementary Table 1. Health-related fitness in healthy women and affected women with low fatigued and fatigued.

	**Control Group (*n* = 25)**	**ENDO Group (*n* = 25)**		***p*-trend**
		**Low Fatigue (*n* = 13)**	**Fatigued (*n* = 12)**	
Dynamometry				
Back dynamometer	63.20 ± 11.40	53.33 ± 12.21	51.03 ± 17.43	0.004
	58.26–68.12)	45.95–60.71)	39.95–62.10	
Hand dynamometer, dominant side	26.50 ± 4.00	26.15 ± 4.92	25.89 ± 4.69	0.688
	24.53–27.46)	23.38–29.32	22.90–28.87	
Hand dynamometer, nondominant side	25.20 ± 3.90	23.59 ± 5.65	24.67 ± 4.40	0.470
	23.35–26.01)	20.17–27.00	21.88–27.47	
Lumbar spine flexibility				
Schöeber test (cm)	15.00 ± 1.09	14.31 ± 0.93	13.96 ± 1.20	0.008
	14.48–15.46)	13.75–14.87)	13.20–14.72	
Flamingo test	0.02 ± 0.10	0.19 ± 0.33	0.17 ± 0.33	0.039
	−0,02–0.07)	0.00–0.39	−0.04–0.37	
6-min walking test (m)	651.20 ± 54.30	571.85 ± 65.51	549.75 ± 48.50	<0.001
	624.42–672.71)	532.26–611.43	518.94–580.56	

Values are expressed as mean ± standard deviation (95% confidence intervals for the mean).

Supplementary Table 2. Perceived physical fitness, sleep quality and quality of life in healthy women and affected women with low fatigued and fatigued.

	**Control Group** *(n = 25)*	**ENDO Group** *(n = 25)*		***p*-trend**
		**Low Fatigue** *(n = 13)*	**Fatigued** *(n = 12)*	
Physical fitness				
Overall fitness	3.60 ± 0.65	3.08 ± 0.64	2.30 ± 0.82	0.018
	3.31–3.86	2.69–3.46	1.71–2.89	
Cardio-respiratory fitness	3.25 ± 0.79	2.85 ± 0.80	2.20 ± 0.92	0.087
	2.91–3.59	2.36–3.33	1.54–2.86	
Muscular fitness	3.63 ± 0.71	3.5 ± 0.5	2.6 ± 1.0	0.018
	3.32–3.93	3.15–3.78	1.91–3.29	
Speed-agility	3.71 ± 0.75	3.15 ± 0.38	2.70 ± 0.67	0.051
	3.39–4.03	2.93–3.38	2.22–3.18	
Flexibility	3.38 ± 0.77	3.15 ± 1.07	3.10 ± 1.10	0.870
	3.05 –3.70	2.51–3.80	2.31–3.89	
Sleep quality	6.00 ± 3.19	6.70 ± 3.47	10.44 ± 3.68	0.014
	4.68–7,32	4.22–9.18	7.62–13.27	
HRQoL				
Physical health	55.09 ± 5.04	44.24 ± 10.0	31.04 ± 13.02	0.034
	53.01–57.17	37.09–51.39	21.03–41.05	
Mental health	49.56 ± 10.54	49.40 ± 9.29	43.00 ± 13.59	0.327
	45.21–53.91	42.75–56.04	32.55–53.44	

Values are expressed as mean ± standard deviation (95% confidence intervals for the mean).
